# Influence of crystallinity on the thermoelectric power factor of P3HT vapour-doped with F4TCNQ

**DOI:** 10.1039/c7ra11912g

**Published:** 2018-01-04

**Authors:** Jonna Hynynen, David Kiefer, Christian Müller

**Affiliations:** Department of Chemistry and Chemical Engineering, Chalmers University of Technology 41296 Göteborg Sweden christian.muller@chalmers.se

## Abstract

Doping of the conjugated polymer poly(3-hexylthiophene) (P3HT) with the p-dopant 2,3,5,6-tetrafluoro-7,7,8,8-tetracyanoquinodimethane (F4TCNQ) is a widely used model system for organic thermoelectrics. We here study how the crystalline order influences the Seebeck coefficient of P3HT films doped with F4TCNQ from the vapour phase, which leads to a similar number of F4TCNQ anions and hence (bound + mobile) charge carriers of about 2 × 10^−4^ mol cm^−3^. We find that the Seebeck coefficient first slightly increases with the degree of order, but then again decreases for the most crystalline P3HT films. We assign this behaviour to the introduction of new states in the bandgap due to planarisation of polymer chains, and an increase in the number of mobile charge carriers, respectively. Overall, the Seebeck coefficient varies between about 40 to 60 μV K^−1^. In contrast, the electrical conductivity steadily increases with the degree of order, reaching a value of more than 10 S cm^−1^, which we explain with the pronounced influence of the semi-crystalline nanostructure on the charge-carrier mobility. Overall, the thermoelectric power factor of F4TCNQ vapour-doped P3HT increases by one order of magnitude, and adopts a value of about 3 μW m^−1^ K^−2^ in the case of the highest degree of crystalline order.

## Abstract 2

The crystallinity of P3HT strongly benefits the electrical conductivity but not Seebeck coefficient, leading to an increase in power factor by one order of magnitude.

## Graphical abstract

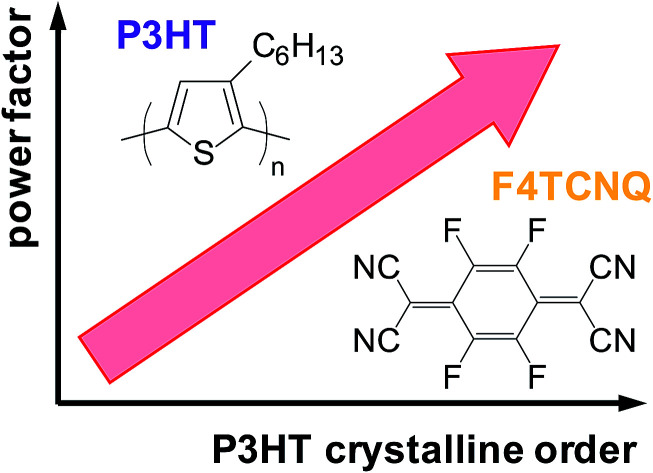

## Introduction

The interest in organic thermoelectrics is rapidly increasing because it may meet the demand for cheap autonomous power sources that will be needed to run countless electronic devices such as sensors, actuators and identification tags, which are envisaged to make up tomorrow's Internet of Things.^1–3^ The ability of different materials to turn heat into electricity can be compared based on their thermoelectric figure of merit:1
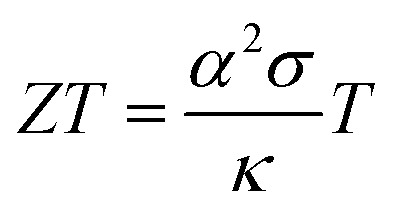
where *α* is the Seebeck coefficient, *σ* the electrical conductivity, *κ* the thermal conductivity, and *T* the absolute temperature. For most organic materials, the thermal conductivity is low even at the highest achievable doping levels, *i.e. κ* < 1 W m^−1^ K^−1^.^4,5^ Hence, to a good approximation the thermoelectric efficacy can be compared based on the power factor *α*^2^*σ*. The Seebeck coefficient and the electrical conductivity are interrelated, and the highest power factor is typically obtained for the most conducting material. Increasing the number of charge carriers usually leads to an increase in electrical conductivity but a decrease in Seebeck coefficient, which scale according to:^6^2
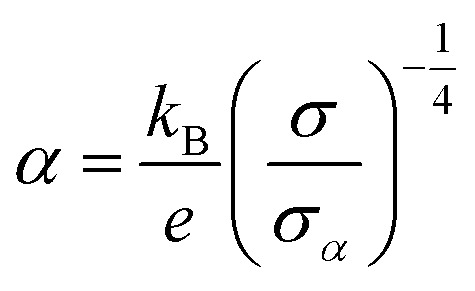
where 
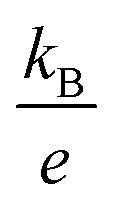
 is the Boltzmann constant divided by unit charge, or the natural unit of thermopower 86.17 μV K^−1^, and *σ*_*α*_ is a free parameter set to 1 S cm^−1^.

**Fig. 1:**
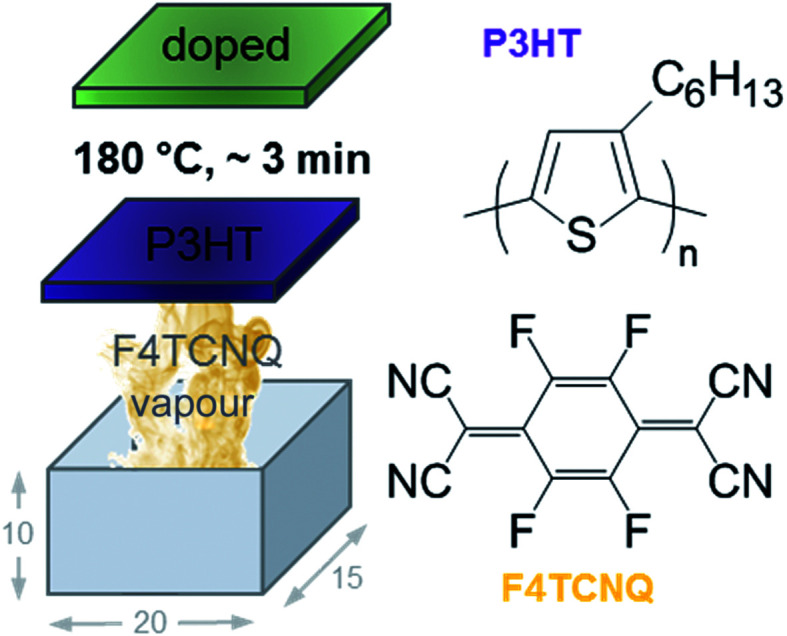
Schematic of home-built vapour doping chamber with the dimensions 20 × 15 × 10 mm; chemical structure of P3HT and F4TCNQ; schematic reproduced with permission from ref. 18; copyright the American Chemical Society 2017.

Conjugated polymers are of particular interest because a wide range of rheological and mechanical properties can be selected through careful choice of the molecular weight. Further, many conjugated polymers now offer a high charge-carrier mobility *μ*, which is needed to reach a high electrical conductivity according to:

3 *σ* = *nqμ*

where *n* is the number of charge carriers and *q* is their charge, *i.e.* ±1.6 × 10^−19^ C for electrons and holes. The charge carrier density can be increased through doping, either by acid doping or redox doping.^3^ The latter can be conveniently carried out by adding a so-called molecular dopant to the conjugated polymer. The dopant molecule then either accepts or donates an electron from/to the conjugated polymer, which gives rise to p- or n-doping, and leads to the formation of a charge transfer complex (partial charge transfer) or ion pair (integer charge transfer). p-type doping of poly(3-hexylthiophene) (P3HT) with 2,3,5,6-tetrafluoro-7,7,8,8-tetracyanoquinodimethane (F4TCNQ) is a widely used model system (Fig. 1),^7–9^ where an electron is donated from the highest occupied molecular orbital (HOMO) of P3HT to the lowest unoccupied molecular orbital (LUMO) of F4TCNQ, leading to integer charge transfer.^8,10^

The dopant can be introduced by two means, either through co-processing together with the polymer from the same solution,^6,7,11–13^ which leads to aggregation that disrupts the solid-state order of P3HT,^14^ or through sequential doping.^14–17^ Sequential doping can be carried out by either depositing the dopant onto the polymer from the vapour phase (‘vapour doping’),^15,18^ or by bringing the polymer in contact with an orthogonal solvent that dissolves the dopant.^14,16,17^ Sequential doping is of interest since it allows to circumvent the aggregation of P3HT that occurs during co-processing. Instead, the nanostructure of the polymer can develop under controlled conditions. Subsequently, P3HT can be doped in a precise manner, which allows to study the interplay of charge-carrier density, nanostructure and electrical properties.^17–19^ Moreover, sequential doping can lead to a significantly higher electrical conductivity above 10 S cm^−1^.^17,18^

Recently, we as well as others have studied the influence of the crystallinity on the electrical conductivity of sequentially doped P3HT.^18–20^ By tuning the regioregularity and processing solvent the nanostructure of P3HT could be altered leading to a much higher electrical conductivity. For example, by changing the polymer processing solvent we were able to increase the electrical conductivity from 0.01 to 13 S cm^−1^, which we attributed to the higher charge-carrier mobility that resulted from a higher degree of polymer crystallinity.^18^ Similar findings were made by Scholes *et al.*^19^ and Chew *et al.*^20^ who concluded that the ordered regions of P3HT give rise to a higher charge-carrier mobility and therefore lead to an increase in the electrical conductivity. Here, we explore how the crystallinity influences the power factor of P3HT vapour-doped with F4TCNQ. We find that an increase in the crystalline order enhances the power factor by one order of magnitude from 0.2 to 2.7 μW m^−1^ K^−2^.

## Results and discussion

To vary the crystalline order of P3HT we spincoated films with a thickness of ∼70 nm at 60 °C from six different solvents (chloroform, chlorobenzene, chlorobenzene/*o*-dichlorobenzene, toluene, 1,2,4-trichlorobenzene and *p*-xylene). We chose to work with a highly regioregular P3HT that featured a number-average molecular weight of *M*_n_ ∼ 29 kg mol^−1^.

**Fig. 2:**
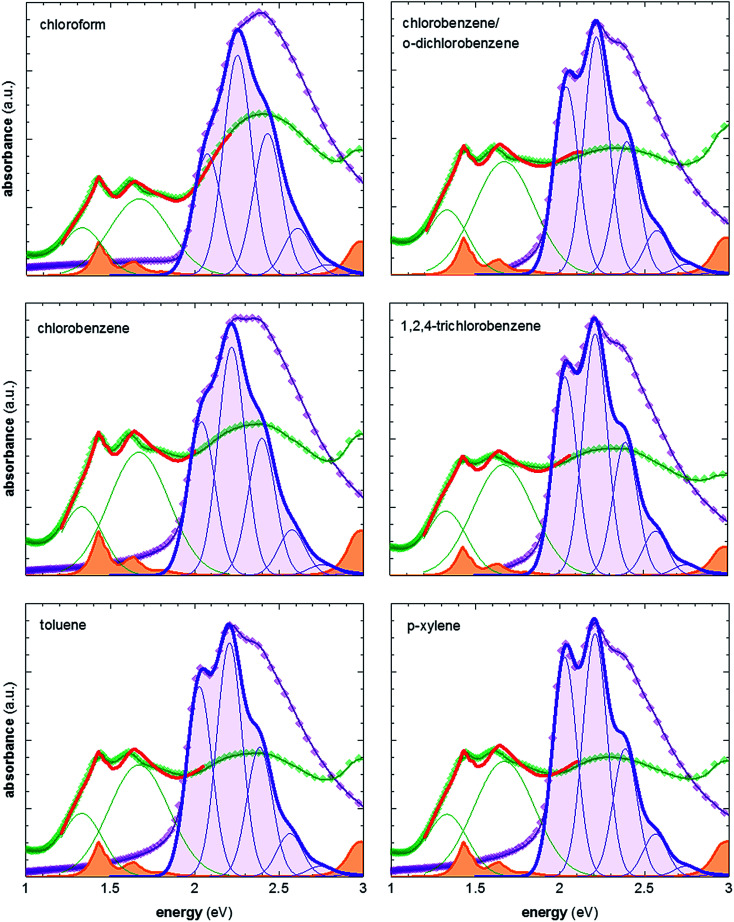
Representative UV-vis absorption spectra of neat P3HT films spincoated from various solvents at 60 °C (purple symbols), and films vapour doped for 3 min (green symbols); spectra of neat P3HT are fitted according to ref. 21–23; spectra of doped P3HT are fitted according to ref. 11 and 24, and are composed of (1) two Gaussians representing the contribution from polaron absorption centered at 1.33 eV and 1.67 eV, respectively (green), (2) P3HT aggregate absorption modelled according to ref. 21–23 (not shown), and (3) measured absorption spectrum of the F4TCNQ anion (orange) ref. 24.

To compare the degree of solid-state order of neat P3HT films, we recorded UV-vis absorbance spectra. We fitted the spectra according to the model developed by Spano *et al.*, assuming a Huang–Rhys factor of 1:^21–23^4
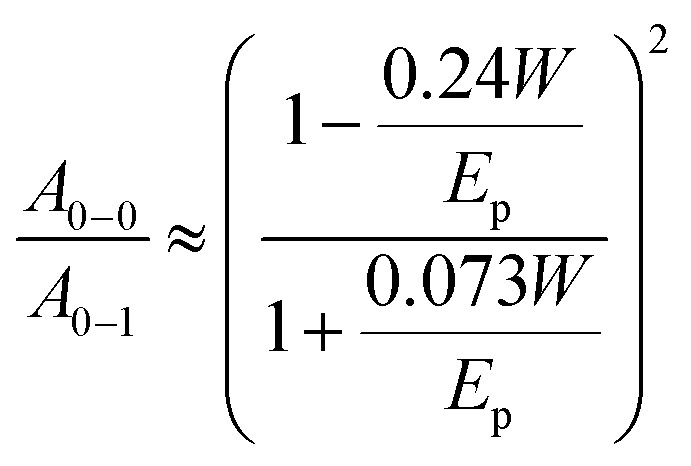
where *E*_p_ is the intramolecular vibration (0.18 eV) and the 
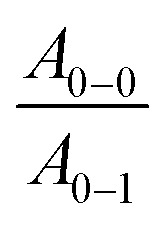
 ratio is taken from the absorption spectra. We used eqn (4) to extract the free exciton bandwidth *W*, which we used as a measure for the degree of crystallinity. The free exciton bandwidth varied from 155 meV to 30 meV for chloroform and *p*-xylene, respectively, indicating the highest degree of order in case of the latter (Fig. 2).

**Fig. 3:**
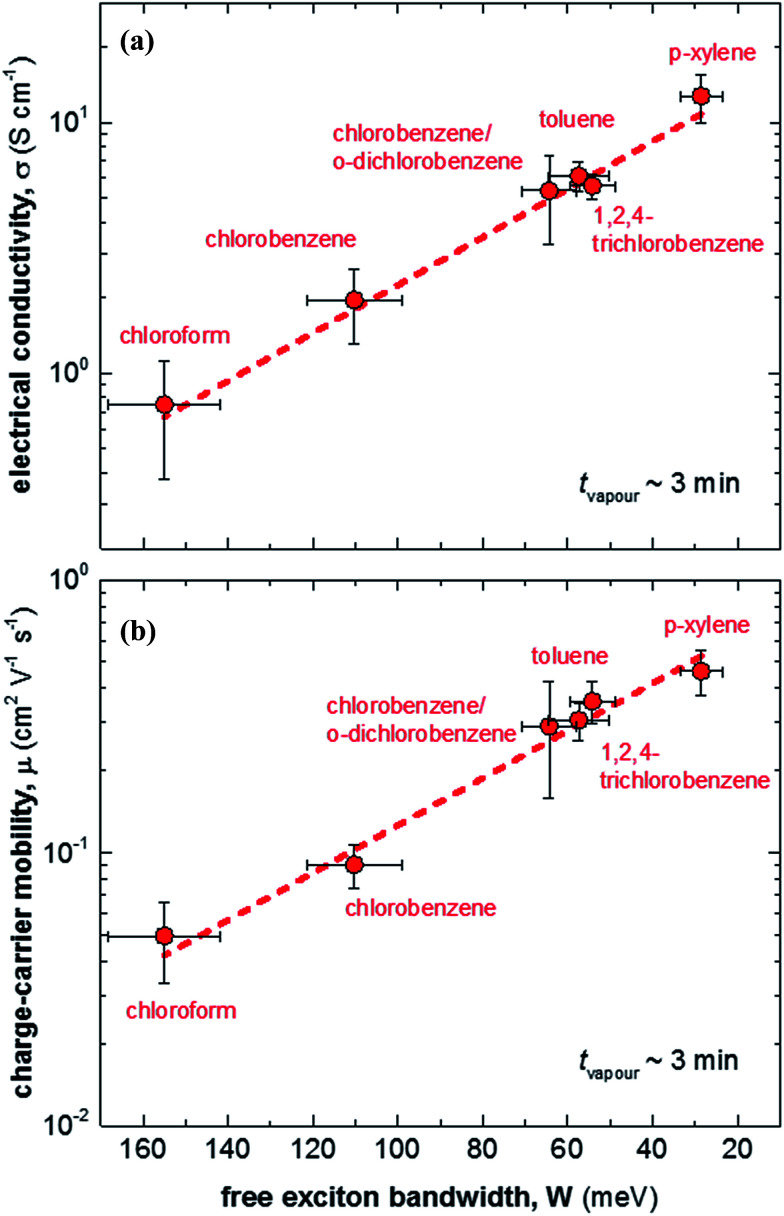
(a) Electrical conductivity *σ* as a function of free exciton bandwidth *W*, calculated by fitting UV-vis spectra according to ref. 21–23: (b) charge-carrier mobility μ as a function of free exciton bandwidth *W*.

We then doped the neat P3HT films by exposing them to vapour of F4TCNQ at ambient conditions as previously described (for vapour doping setup see Fig. 1).^18^ The degree of doping correlates with the period of time that the samples are exposed to F4TCNQ vapour. For the vapour-doped samples, we find that the conductivity sharply increases during the first two minutes of doping but then levels off for longer doping times (Table 1). We therefore selected a doping time of 3 min to compare samples spincoated from different solvents. For the least ordered samples spincoated from chloroform we find an electrical conductivity of only 0.7 S cm^−1^, whereas more highly ordered *p*-xylene films yielded a value of 12.7 S cm^−1^ after 3 min of doping. We have demonstrated previously that the electrical conductivity increases with the degree of crystalline order of P3HT, as evidenced by the inverse correlation of *σ* and *W* (Fig. 3).^18^

**Table 1:** Electrical conductivity *σ* and Seebeck coefficient *α* as a function of vapour doping time *t*_vapour_ for films spincoated from chlorobenzene/*o*-dichlorobenzene, and *p*-xylene

Solvent	*t*_vapour_ (min)	*σ* (S cm^−1^)	*α* (μV K^−1^)
Chlorobenzene/*o*-dichlorobenzene	0.5–2	0.6 ± 1.3	62 ± 17
	2.5–5	5.5 ± 2.0	54 ± 4
	10	2.9 ± 2.3	57 ± 4
*p*-Xylene	0.5–2	5.0 ± 5.4	54 ± 11
	2.5–5	12.7 ± 2.8	43 ± 3
	10	14.5 ± 0.5	48 ± 2

We explain the correlation between conductivity and crystallinity with the well-established impact of the former on the charge-carrier mobility. We can use eqn (3) to estimate the mobility if we can determine the number of charge carriers. Since doping of P3HT with F4TCNQ occurs through integer charge transfer, we assume that each F4TCNQ anion corresponds to one charge on the polymer. We here do not distinguish between bound and free charge carriers and instead consider the average mobility. Therefore, we equate the F4TCNQ anion concentration with the number of charge carriers. We estimate the anion concentration by fitting UV-vis spectra of doped films according to a procedure first proposed by Wang *et al.*^24^ For all six processing solvents we extract a similar value of about 2 × 10^−4^ mol cm^−3^, which corresponds to 0.03 anions per P3HT repeat unit (or 4.7 wt% anions assuming a density of 1 g cm^−3^). Based on this value we find that for the here studied samples the mobility increases from 0.05 to 0.5 cm^2^ V^−1^ s^−1^ (Fig. 3). Pingel *et al.* have shown for F4TCNQ-doped P3HT that only a fraction of anions gives rise to free charge carriers,^25^ which implies that our mobility estimate represents a lower bound.

The Seebeck coefficient, instead, shows a markedly different, non-monotonic behaviour. For the most disordered samples, spincoated from chloroform, we measure a low Seebeck coefficient of 51 μV K^−1^ (Table 2). For more ordered samples we find a slightly higher value of 60 μV K^−1^, whereas the most ordered samples, spincoated from *p*-xylene, display the lowest Seebeck coefficient of 43 μV K^−1^. In case of poly(3,4-ethylenedioxythiophene):tosylate (PEDOT:Tos) with a constant dopant concentration the Seebeck coefficient has been shown to slightly increase with the degree of crystalline order.^26^ Fabiano *et al.* argue that the slight increase in Seebeck coefficient from 32 to 44 μV K^−1^ arises because of a steeper density of states at the Fermi level, caused by the presence of states in the bandgap as a result of delocalisation in ordered domains. Since the charge-carrier mobility likewise improves through the presence of ordered domains, a correlation between the Seebeck coefficient and mobility is observed for PEDOT:Tos. We argue that a similar behaviour results in the slight increase in Seebeck coefficient that we observe for more disordered samples, *e.g.* when changing the spincoating solvent from chloroform to chlorobenzene (Table 2).

**Table 2:** Calculated values of the free exciton bandwidth *W*, estimated values of aggregate percentage according to ref. 21–23, and measured electrical conductivities and Seebeck coefficients for samples tested in this study

Solvent	*W* (meV)	Aggregates (%)	*σ* (S cm^−1^)	*α* (μV K^−1^)	F4TCNQ anion conc. (10^−4^ mol cm^−3^)
Chloroform	155	33	0.7 ± 0.4	51 ± 2	2.3 ± 0.3
Chlorobenzene	110	38	2.0 ± 0.7	63 ± 1	2.9 ± 0.6
Toluene	58	42	5.3 ± 2.1	55 ± 4	2.1 ± 0.4
Chlorobenzene/*o*-dichlorobenzene	64	42	6.1 ± 0.8	59 ± 3	1.9 ± 0.4
1,2,4-Trichlorobenzene	54	43	5.6 ± 0.6	56 ± 2	1.7 ± 0.3
*p*-Xylene	30	46	12.7 ± 2.8	46 ± 2	3.0 ± 0.6

The slight decrease in Seebeck coefficient that we observe for more ordered samples may arise because of an increase in the number of mobile charge carriers. The Seebeck coefficient predominately probes mobile charge carriers and therefore a change in their concentration will influence the measured thermovoltage. We note that the concentration of F4TCNQ anions does not change with the degree of order. Therefore, we deem it likely that the same number of charges are generated per volume of amorphous and crystalline phase. However, the mobility of charges may depend on the local order in the vicinity where a particular charge is generated. Gao *et al.* have argued that both regiorandom and regioregular P3HT can interact with F4TCNQ when dissolved in a common solvent.^27^ However, only in case of regioregular P3HT free charges are generated because the polymer is readily able to adopt a planar conformation, which facilitates delocalisation of hole charges. In our samples, both disordered, amorphous and ordered, crystalline domains are present. Doping of P3HT in crystalline domains readily leads to a free charge because the polymer is already planarised. Instead, doping of P3HT in amorphous domains requires that the polymer chain adopts a more planar conformation upon doping. Consistent with this picture, Chew *et al.* have recently proposed that for sequentially doped P3HT the film connectivity is improved because doping leads to more extended P3HT crystallites.^20^ We argue that molecular motion in amorphous domains is more restricted than in a dilute polymer solution, discussed in the work by Gao *et al.*,^27^ and therefore doping cannot readily induce the same degree of order that is already present in crystalline domains. The presence of structural defects implies that at least some charges are bound. Hence, we anticipate that the amount of mobile charges increases with the initial crystallinity of P3HT, leading to a reduction of the Seebeck coefficient, which may explain the slightly lower value that we measured for the most ordered samples spincoated from *p*-xylene.

**Fig. 4:**
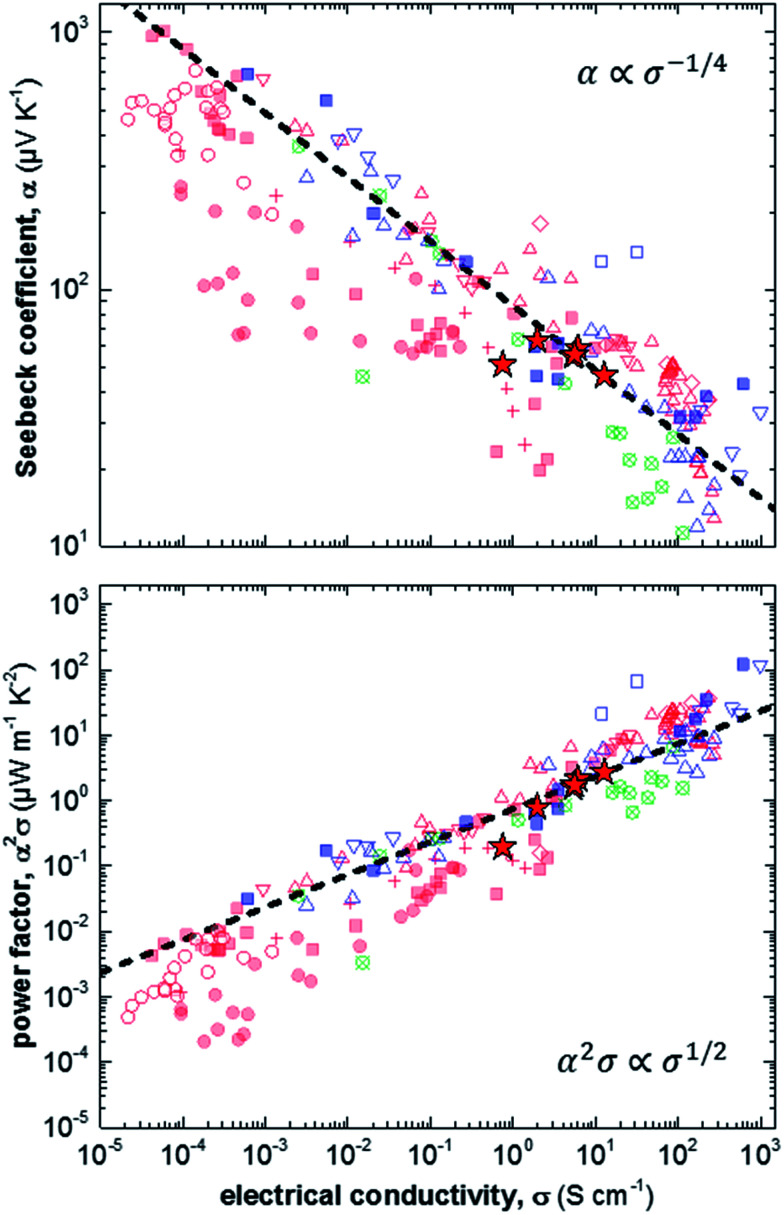
Seebeck coefficient *α* (top) and power factor *α*^2^*σ* (bottom) as a function of electrical conductivity *σ* measured in this study (red stars), and extracted from literature: P3HT doped with F4TCNQ (filled red squares),^6,17,28^ NOPF_6_ (red crosses),^29^ FTS or TFSI (open red triangles),^6,30–32^ or FeCl_3_ (open red diamonds);^33^ P3HT:PEO doped with F4TCNQ (filled red circles);^11^ P3HT:P3HTT doped with F4TCNQ (open red circles);^34^ PBTTT doped with F4TCNQ (filled blue squares),^6,35^ FTS or TFSI (open blue triangles),^6,30,36^ or F2TCNQ (open blue squares);^35^ and p(g_4_2T-T) doped with F4TCNQ or DDQ (open crossed circles);^37^ the dashed lines are drawn according to eqn (2) [FTS = (tridecafluoro-1,1,2,2-tetrahydrooctyl)trichlorosilane; TFSI = Fe(iii)triflate; P3HTT = poly(3-hexylthiothiophene); PBTTT = poly(2,5-bis(3-tetradecylthiophen-2-yl)thieno[3,2-*b*]thiophene); F2TCNQ = 2,5-difluoro-7,7,8,8-tetracyanoquinodimethane; DDQ = 2,3-dichloro-5,6-dicyano-*p*-benzoquinone].

Finally, we compare our results with other reports for a number of different polythiophenes, including P3HT, the copolymer poly(bithiophene-*alt*-thienothiophene) (PBTTT) and the oligo ethylene glycol bearing derivative p(g_4_2T-T) (Fig. 4).^6,11,17,28–37^ The variation in Seebeck coefficient with electrical conductivity follows the empirical trend described by eqn (2) (Fig. 4, top). Data points that lie to the left of this empirical line are thought to be mobility limited: a given degree of doping results in a certain Seebeck coefficient, but many charges cannot traverse the material sufficiently quickly because their motion is impeded by structural defects. One illustrative example are ternary blends of P3HT:F4TCNQ with poly(ethylene oxide) (PEO). We have found that in case of a sufficiently high concentration of P3HT:F4TCNQ the Seebeck coefficient and electrical conductivity follow the empirical trend of eqn (2).^11^ In contrast, in case of more dilute blends the connectivity between P3HT:F4TCNQ domains is poor, leading to a lower mobility and hence electrical conductivity (*cf.* red filled circles in Fig. 4). We note that the conductivity that we have measured for samples spincoated from chloroform diverges from the empirical trend of eqn (2). We assign this behaviour to the low charge-carrier mobility that we deduce for these samples, caused by poor connectivity of crystalline domains.

The opposite case are samples that are mobility enhanced, where charges are able to traverse the material more quickly than predicted by the empirical trend of eqn (2), leading to a higher conductivity for a given Seebeck coefficient. An example is the recent work by Brinkmann *et al.*, who studied sequentially-doped P3HT films that had been uniaxially aligned through rubbing.^17^ Upon F4TCNQ doping a concomitant increase in electrical conductivity and Seebeck coefficient was observed, with maximum values of *σ* = 22 S cm^−1^ for *α* = 60 μV K^−1^, which we rationalise with improved connectivity along the direction of orientation.

## Conclusions

**Fig. 5:**
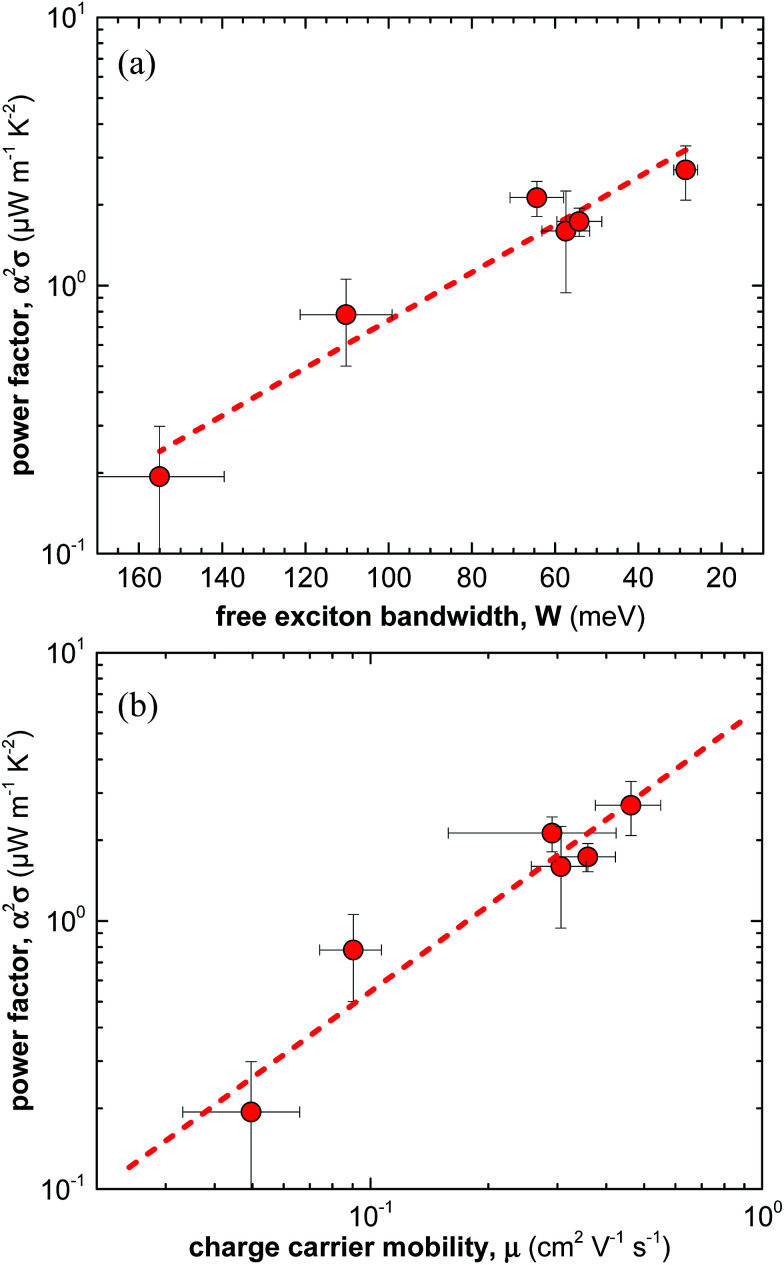
(a) Power factor *α*^2^*σ* as a function of free exciton bandwidth *W* calculated by fitting UV-vis spectra according to ref. 21–23, and (b) power factor *α*^2^*σ* as a function of average charge-carrier mobility *μ*.

Overall, for the here studied F4TCNQ vapour-doped P3HT thin films we find that the thermoelectric power factor *α*^2^*σ* increases with the degree of crystalline order from 0.2 to 2.7 μW m^−1^ K^−2^ (Fig. 5a). We ascribe this behaviour to improved charge-carrier mobility and hence electrical conductivity (Fig. 5b). In contrast, we only observe small changes in Seebeck coefficient. Our work indicates that efforts to improve the thermoelectric power factor of conjugated polymer based materials should primarily focus on enhancing the electrical conductivity. For instance, this could be achieved by selecting processing protocols that lead to a for charge transport beneficial nanostructure, *i.e.* a nanostructure that gives rise to a high charge-carrier mobility.

## Experimental

### Materials

P3HT was obtained from Ossila Ltd. (regioregularity ∼ 96%, number-average molecular weight of *M*_n_ ∼ 29 kg mol^−1^, polydispersity index ∼ 2.2). The regioregularity was determined with a 475 Agilent (Varian) MR 400 MHz spectrometer with CDCl_3_ as the solvent. The molecular weight was measured with size exclusion chromatography (SEC) on an Agilent PL-GPC 220 integrated high temperature GPC/SEC system in 1,2,4-trichlorobenzene at 150 °C using relative calibration with polystyrene standards. F4TCNQ was purchased from TCI Chemicals and used without further purification. Solvents with purity >99% were purchased from Sigma-Aldrich (*o*-dichlorobenzene, chlorobenzene, *p*-xylene, 1,2,4-trichlorobenzene) and Fisher Scientific (chloroform, toluene).

### Sample preparation and vapour doping

P3HT was dissolved at 60 °C at a concentration of 10 g l^−1^ in various solvents. Thin films were spincoated from 60 °C hot solutions onto cleaned glass substrates. Substrates were cleaned with soapy water then in a sonication bath; first with acetone (15 min) then with iso-propanol (15 min) and finally dried with nitrogen. All solutions were spin coated for 60 s at 1000 rpm, followed by 30 s at 3000 rpm. The thickness of spincoated films was determined using a KLA Tencor AlphaStep D-100 profilometer. F4TCNQ was thermally evaporated onto P3HT thin films at ambient pressure using a home-built evaporation chamber that consisted of a 15 × 20 mm large glass compartment in which films were suspended upside down, 10 mm above a crucible that contained ∼20 mg of F4TCNQ. The crucible was heated to a temperature of 180 °C during doping on a hotplate and a stainless-steel block was placed on top of the P3HT thin film to act as a heat sink to avoid thermal degradation of the polymer. The film temperature was measured with a handheld temperature probe attached to the glass slide and reached a maximum of 60 °C.

### UV-vis spectroscopy

Measurements were performed with a PerkinElmer Lambda 900 spectrophotometer. Absorption spectra of neat P3HT were fitted according to ref. 21–23. Absorption spectra of F4TCNQ-doped P3HT were fitted according to ref. 11 and 24 using a superposition of two Gaussian peaks (centred at 1.33 and 1.67 eV; FWHM of 0.29 and 0.42 eV, respectively), a P3HT aggregate model ref. 21–23, and the F4TCNQ anion spectrum from ref. 11 and 24.

### Electrical characterisation

The electrical resistivity was measured with a four-point probe setup from Jandel Engineering (cylindrical probe head, RM3000) using collinear tungsten carbide electrodes with equidistant spacing of 1 mm that were held down with a constant weight of 60 g. Seebeck coefficients were measured at 300 K with an SB1000 instrument equipped with a K2000 temperature controller from MMR Technologies using a thermal load of 1–2 K and a constantan wire as an internal reference. Samples of about 1 mm times 4 mm were mounted on the sample stage using silver paint (Agar Silver Paint, G302).

## Conflicts of interest

There are no conflicts to declare.

## Supplementary Material
